# Protective effects of (-)-epigallocatechin gallate on UVA-induced damage in ARPE19 cells

**Published:** 2008-12-30

**Authors:** Chi-Ming Chan, Jheng-Hua Huang, Hsin-Huang Lin, Han-Sun Chiang, Bing-Huei Chen, Jing-Yin Hong, Chi-Feng Hung

**Affiliations:** 1 School of Medicine, Fu Jen Catholic University, Taipei Hsien, Taiwan; 2 Department of Ophthalmology, Cardinal Tien Hospital, Taipei Hsien, Taiwan; 3 Department of Internal Medicine, Cathay General Hospital, Taipei, Taiwan; 4 Graduate Institute of Basic Medicine, Fu Jen University, Taipei Hsien, Taiwan; 5 Department of Life Science, Fu-Jen Catholic University, Taipei Hsien, Taiwan

## Abstract

**Purpose:**

Oxidative injury to the retinal pigment epithelium (RPE) has been proposed to play a contributing role in age-related macular degeneration (AMD). Exposure to solar ultraviolet (UV) radiation is believed to cause the production of reactive oxygen species (ROS), which may cause oxidative damage to RPE cells. Studies have shown that (-)-epigallocatechin gallate (EGCG), an abundant and active component in green tea, can protect several cell types from oxidative stress. It may be useful in the prevention of early AMD.

**Methods:**

To determine whether EGCG protects RPE cells from UVA-induced damage, we used a cell viability assay to determine the viability of UVA-treated cells. Intracellular H_2_O_2_ levels were measured by flow cytometry. Western blotting was used to detect UVA-induced signaling pathways.

**Results:**

The results indicated that EGCG inhibits UVA-induced RPE cell death. In addition, intracellular H_2_O_2_ generation in RPE cells irradiated by UVA was inhibited by EGCG in a concentration-dependent manner. EGCG also inhibited UVA-induced extracullar signal-regulated kinase (ERK) and c-jun-NH2 terminal kinase (JNK) activation in RPE cells while a higher concentration of EGCG had an inhibitory effect on UVA-induced p38 activation. Finally, we investigated cyclooxygenase-2 (COX-2) expression in RPE cells exposed to UVA radiation, and EGCG was found to also have inhibited UVA-induced COX-2 expression.

**Conclusions:**

Taken together, our results demonstrate that EGCG inhibits UVA-induced H_2_O_2_ production, mitogen-activating protein kinase activation, and expression of COX-2. Moreover, it enhances RPE cell survival after UVA exposure. This suggests EGCG is effective in preventing UVA-induced damage in RPE cells and may be suitable for further developments as a chemoprotective factor for the primary prevention of early AMD.

## Introduction

Age-related macular degeneration (AMD) is one of the most common causes of severe visual loss in the elderly population in the developed world [1,2]. Oxidative damage is thought to play an important role in the pathogenesis of AMD. In the tissues, the impairment of retinal pigment epithelium (RPE) cell function is an early and crucial event in the molecular pathways leading to AMD [3,4].

Ultraviolet (UV) radiation is part of the sunlight spectrum. Only ultraviolet B (UVB; 280–320 nm) and ultraviolet A (UVA; 320–400 nm) reach the terrestrial surface [5]. Exposure to solar UV radiation has been implicated in some skin and ocular pathologies. UV radiation can induce DNA breakdown and cause the production of reactive oxygen species (ROS). In turn, ROS generated by UV radiation may cause oxidative damage to RPE cells [6-8]. The cornea, aqueous fluid, the adult human lens, and vitreous body filter out most of the ultraviolet radiation below 400 nm, and only transmit visible light radiation. The very young human lens transmits a small window of UV light (approximately 320 nm) to the retina, while the elderly lens blocks much of the short blue visible light (400–500 nm) [9]. However, ozone layer depletion, together with the increase in outdoor recreation and average life span, may give rise to an increase in the accumulative lifetime exposure of the retina to UV radiation [10]. Also, substantial increase in UV radiation reaching the retina may result from the removal of the crystallin lens by cataract surgery [11]. A recent study showed some UV and blue-light filtering intraocular lenses (IOLs) still lack adequate UVA protection [12], while some IOLs show transmittance rates of more than 10% in the range between 350 nm and 400 nm, resulting in insufficient UVA protection [13].

Human cell lines often do not retain the characteristics of the original source cells, especially if they are “immortal.” However, the human ARPE19 cell line has been used for many in vitro studies of UV-induced [14,15] as well as blue-light-induced damage [16,17] to the retina.

Green tea has been shown to have antioxidant and antiinflammatory effects on various types of cells [18,19]. The green tea extracts contain (-)-epigallocatechin gallate (EGCG), (-)-epigallocatechin (EGC), (-)-epicatechin gallate (ECG), (-)-epicatechin (EC), and catechin. Among these constituents, EGCG is the most abundant and the most active component in green tea. Although studies have shown that EGCG protects several cell types from oxidative stress [20-22], there are few studies on the protective effects of EGCG against UVA-induced damage and the precise transduction mechanism of the pathological conditions of the retina.

In this study, we propose that EGCG can protect RPE cells from UVA-induced cell death through its antioxidative effect. We investigated the protective effects of EGCG against UVA-induced RPE cell death and the possible mechanisms involved in RPE cell survival. These mechanisms include the inhibition of the UVA-induced intracellular hydrogen peroxide (H_2_O_2_) production, the mitogen-activated protein kinase (MAPK) activation, and the expression of cyclooxygenase-2 (COX-2).

## Methods

### Materials

EGCG, 3-(4,5-dimethylthiazol-2-yl)-2,5-diphenyltetrazolium bromide (MTT), aprotinin, leupeptin, phenylmethylsulfonyl fluoride (PMSF), sodium fluoride (NaF), and sodium orthovanadate were purchased from Sigma Chemical Co. (St. Louis, MO).

Primary antibodies anti-p38 and anti-phospho- c-jun-NH2 terminal kinase (JNK) were purchased from Cell Signaling Technology (Beverly, MA); anti-JNK, anti- extracullar signal-regulated kinase (ERK)1/2, and anti-phospho-p38 were purchased from R&D System, Inc. (Minneapolis, MN); anti-phospho-ERK1/2, and anti-COX-2 were purchased from Santa Cruz Biotechnology (Santa Cruz, CA). Secondary antibodies, antirabbit-HRP and antigoat-HRP, were also purchased from Santa Cruz Biotechnology. The antibody anti-dihydrorhodamine 123 (DHR 123) was purchased from Molecular Probes (Eugene, OR).

### Cell cultures

Adult human retinal pigment epithelial cells (ARPE19) were purchased from Food Industry Research and Development Institute (Hsinchu, Taiwan). These cells were maintained in Dulbecco’s Modified Eagle’s Medium/Nutrient Mixture F-12 (DMEM/F12) supplemented with 10% fetal calf serum (GibcoBRL, Invitrogen Life Technologies, Carlsbad, CA), 100 units/ml penicillin, and 100 μg/ml streptomycin (Sigma Chemical Co.). The cells were cultured in a humidified incubator at 37 °C and 5% CO_2_. For most of the experiments, cells reaching a 90%–95% of confluence were starved and synchronized in serum-free DMEM for 24 h before they were subjected to further analysis.

### Drug treatment and UVA irradiation

ARPE19 cells were cultured on 24 well plates for cell viability assays, 6 well plates (Costar, Cambridge, MA) for flow cytometric analysis, and in 6 cm culture dishes (Costar) for western blot analysis. Cells were pretreated with various concentrations of EGCG for 2 h. After two washes with DMEM, the cells were then incubated with 300 μl/well, 500 μl/well, 1000 μl/dish phosphate-buffered saline (PBS; 137 mM NaCl, 10 mM Phosphate, 2.7 mM KCl, and pH of 7.4) under UVA irradiation in different trials. UVA irradiation was performed immediately after the DMEM-washes as suggested by the manufacturer. Briefly, the cells were irradiated in a Bio-Sun system illuminator from Vilber Lourmat (Marne-la-Vallée Cedex 1, France). The UVA lamps in the illuminator emit ultraviolet rays between 355 nm and 375 nm, with peak luminosity at 365 nm. UVA radiation was supplied by a closely spaced array of four UVA lamps, which delivered uniform irradiation at a distance of 10 cm. The UVA irradiation dose was 20 J/cm^2^ and took approximately 74–80 min to attain (irradiance: 4.2–4.5 mW/cm^2^). Based on a programmable microprocessor, the Bio-Sun system constantly monitors the UV light emission. The irradiation stops automatically when the energy received matches the programmed energy (range of measure: 0 to 9,999 J/cm^2^). After UVA exposure, the cells were fed with fresh DMEM containing EGCG, incubated for an additional amount of time specified in the following section, and collected for further analysis.

### Cell viability assays

The viability of cells was determined by the MTT. The MTT assay in our laboratory has been previously described [23]. Briefly, PBS- or EGCG-pretreated cells were exposed to UVA and incubated for an additional 24 h. After a brief wash with medium, 0.5 mg/ml MTT in DMEM was used for the quantification of living metabolically active cells [24]. Mitochondrial dehydrogenases metabolized MTT to a purple formazan dye, which was analyzed photometrically at 550 nm. Cell viability was proportional to the absorbance measured [25].

### Flow cytometric analysis of intracellular H_2_O_2_

Intracellular production of H_2_O_2_ was assayed as previously described [23] with minor modifications. Briefly, confluent ARPE19 cells starved with serum-free DMEM were pretreated with various concentrations of EGCG for 2 h. Cells were washed with PBS and DMEM, and then treated with 10 μg/ml DHR 123 in DMEM for 30 min. After a brief wash, the cells were irradiated by UVA and were collected by scraping and centrifugation. The cell pellets were resuspended in 1 ml PBS and then analyzed immediately by the Partec CyFlow ML flow cytometer (Partech GmBH, Munster, Germany) at excitation wavelengths of 488 nm and emission wavelengths of 525 nm. Fluorescence signals of 10,000 cells were collected to calculate the mean fluorescence intensity of a single cell.

### Cell lysate preparation and western blot analysis of JNK, ERK, p38, and COX-2

RPE cells treated with or without UVA irradiation were washed with PBS twice. They were then lysed in radioimmunoprecipitation assay buffer, which contained 17 mM Tris–HCl, pH 7.4, 50 mM NaCl, 5 mM EDTA, 1 mM sodium fluoride, 1% Triton X-100, 1% sodium deoxycholate, 0.1% SDS, 1 mM sodium orthovanadate, 1 mM PMSF, and 1 μg/ml aprotinin and leupeptin (freshly prepared). After sonication, the lysate was centrifuged (14,000x g for 10 min at 4 °C), and the supernatant was removed. The protein content was quantified by a Pierce protein assay kit (Pierce, Rockford, IL). Total protein was separated by electrophoresis on 8% SDS–polyacrylamide gels. The proteins were then electroblotted onto polyvinylidene fluoride (PVDF) membranes and probed using the indicated specific antibodies. Immunoblots were detected by enhanced chemiluminescence (Chemiluminescence Reagent Plus, NEN, Boston, MA). For some of the experiments, the PVDF membrane was stripped at 60 °C for 30 min with a stripping buffer that contained 62.5 mM Tris-HCl, pH 6.7, 2% SDS, and 100 mM β-mercaptoethanol.

### Statistical analysis

Unless otherwise indicated, data are expressed as mean±standard error (SE). Comparison of the mean survival rates of cells under UVA irradiation with and without 10 µM EGCG was made by using the unpaired, two-tailed Student *t*-test. We consider p values <0.05 to be statistically significant. All data were analyzed with SigmaPlot 2002 for Windows (Version 8.00).

## Results

### UVA radiation induces ARPE19 cell death

**Figure 1:**
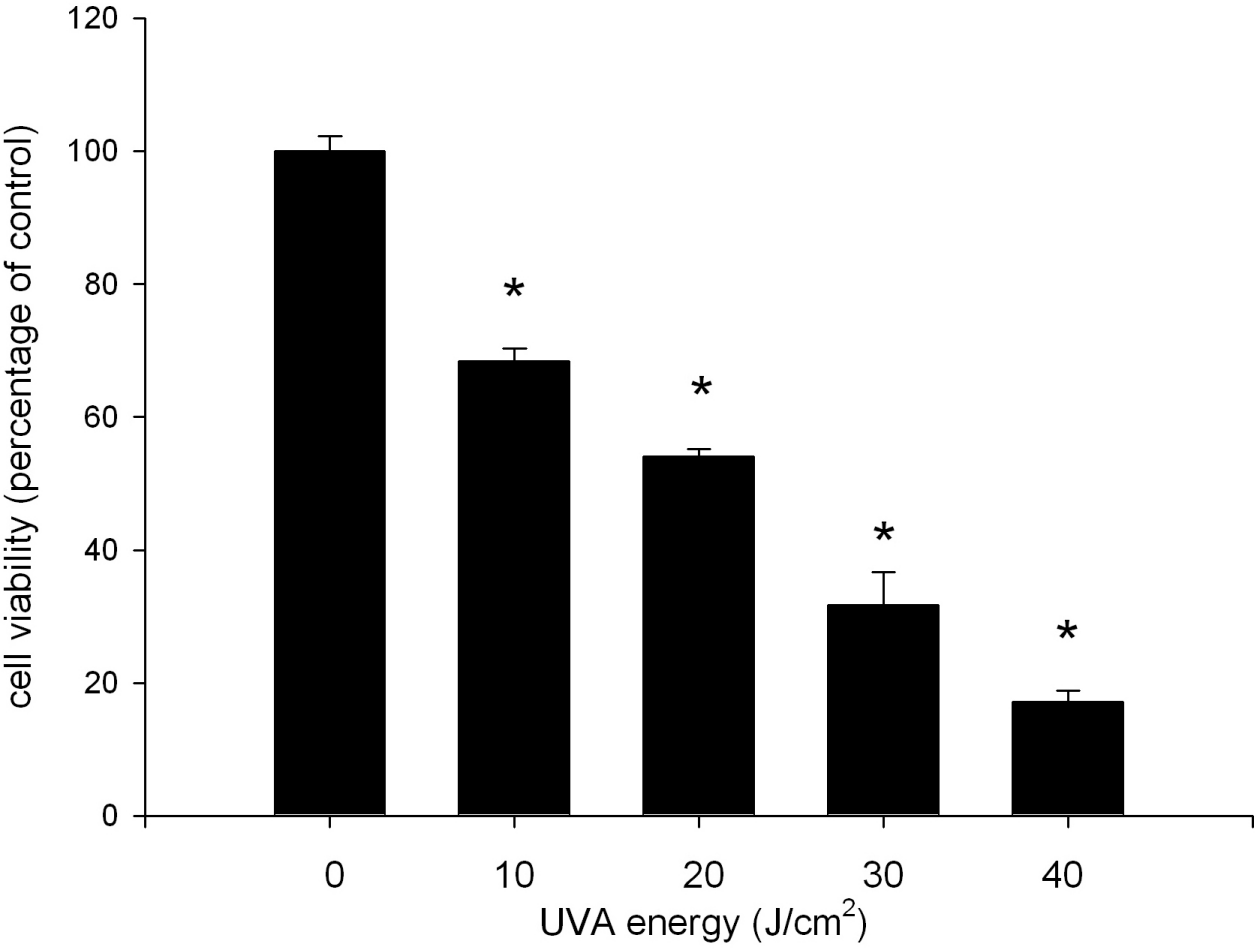
Viability of ARPE19 cells following UVA radiation exposure. The cells were exposed to UVA radiation at indicated doses. They were then incubated in serum-free medium for 24 h. Cell viability was determined by MTT assay. The results are expressed as percentage of control and are represented by mean±standard error (SE) determined from three independent experiments. All p values are for comparisons between control and UVA-irradiated cells. The asterisk indicates p<0.05 versus control.

No available studies had investigated on the cytotoxic effect of UVA radiation on ARPE19 cells. To confirm the cytotoxic effect of UVA irradiation, we exposed cultured ARPE19 cells to increasing doses of UVA radiation (10, 20, 30, and 40 J/cm^2^). Cell viability was determined at 24 h after UVA irradiation. As we expected, ARPE19 cells underwent cell death after UVA exposure in our system. The decrease of cell viability was UVA-dose-dependent, resulting in 68.4, 54.0, 31.7, and 17.2% of remaining survivals at 10, 20, 30, and 40 J/cm^2^ (Figure 1). Since 20 J/cm^2^ of UVA irradiation caused about a half decrease of cell viability (54%), this dosage was used in the following studies on the protective effect on UVA-induced cytotoxicity.

### EGCG has less cytotoxicity on ARPE19 cells

**Figure 2:**
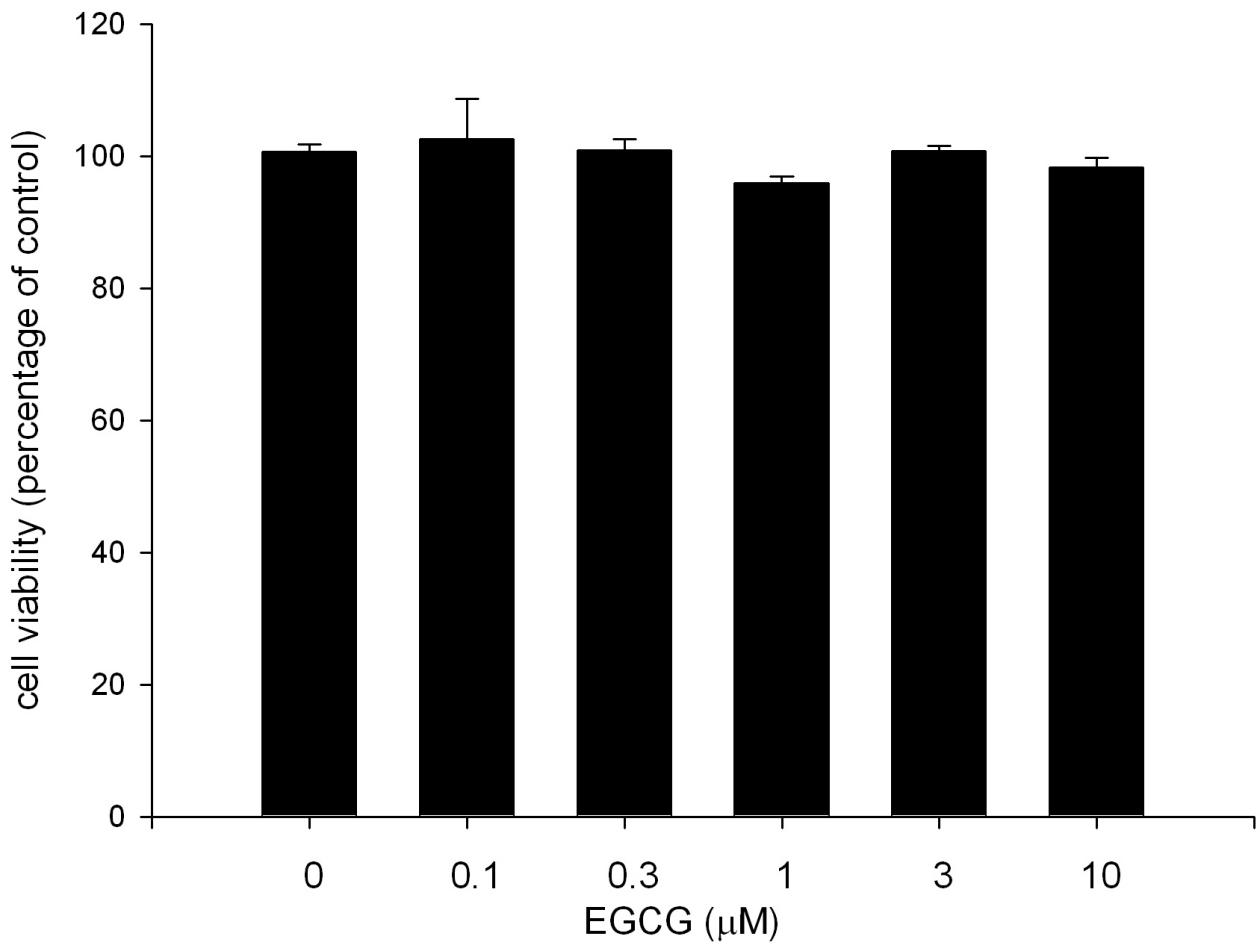
Cytotoxicity of EGCG to ARPE19 cells. ARPE19 cells were treated with or without EGCG for 24 h, and cell viability was assessed by MTT assay. The results are expressed as percentage of control and are represented by mean±SEM (n=4).

To examine whether EGCG produces a toxic effect on ARPE19 cells, we performed a cell viability assay. As shown in Figure 2, EGCG (1–10 μM) treatment did not significantly affect cell viability. The data indicate that EGCG is relatively safe for ARPE19 cells. Therefore 1–10 μM of EGCG were used in the following experiments.

### EGCG inhibits UVA-induced ARPE19 cell death

**Figure 3:**
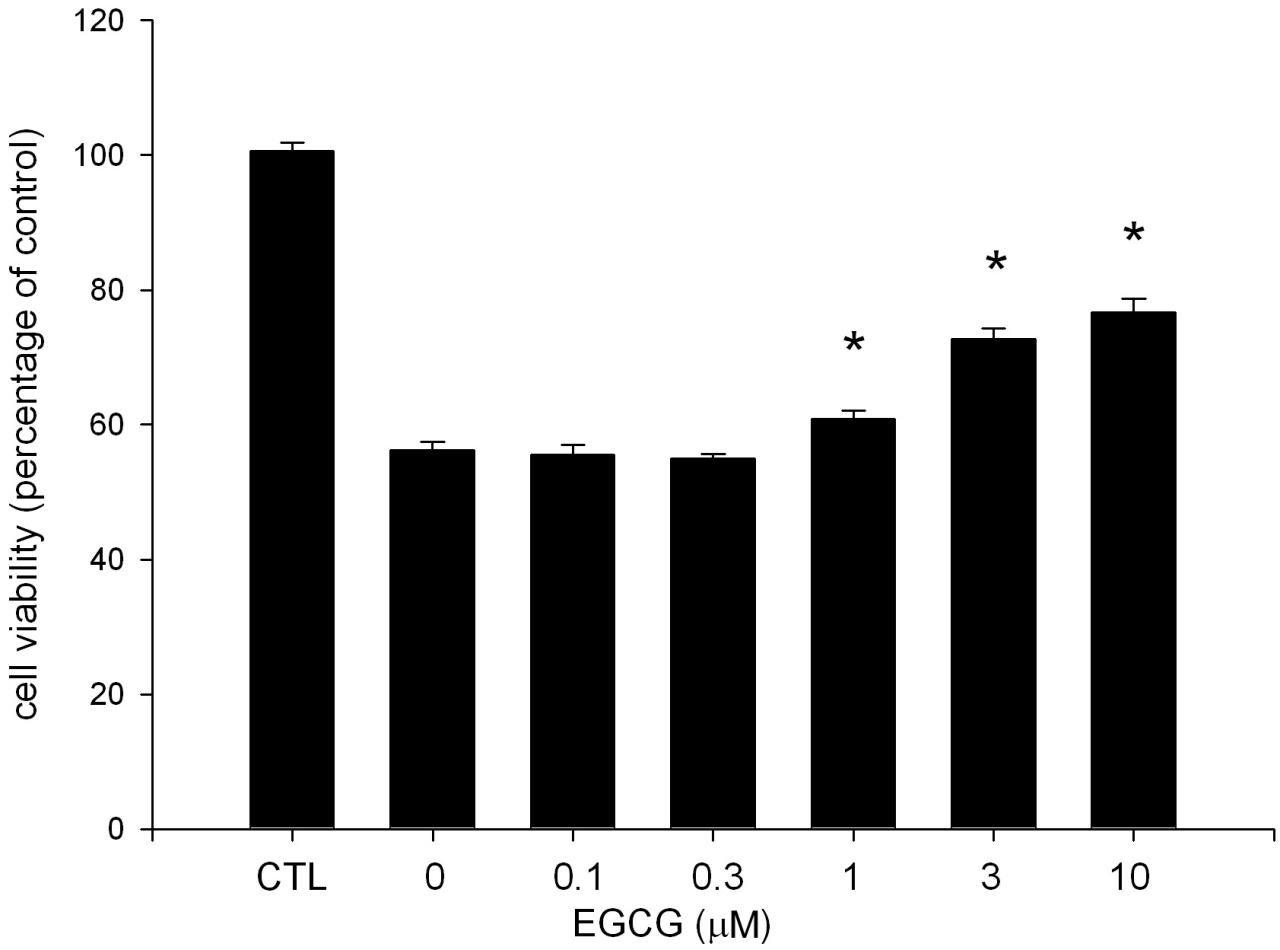
Protective effect of EGCG on ARPE19 cells after UVA radiation exposure. ARPE19 cells were pretreated with EGCG for 2 h before being exposed to UVA radiation. Cell viability was determined by MTT assay 24 h after UVA irradiation (20 J/cm^2^). The control group means the cells were not neither treated with EGCG nor exposed to UVA radiation. The results are expressed as percentage of control and are represented by mean±SEM (n=4). The asterisk indicates p<0.05 versus UVA-exposed cells without EGCG pretreatment.

To determine the protective effects of EGCG on ARPE19 cells, we first performed a cell viability assay. The MTT assay showed that cell viability of ARPE19 cells was decreased after UVA exposure. However, the decrease was reversed by the treatment of EGCG (1, 3 and 10 μM; Figure 3). EGCG at 10 μM produced a marked effect. Approximately 77% of cells were viable upon UVA exposure. The different in cell survival rate under UVA with 10 μM of EGCG and without EGCG was significant (p<0.05). These observations indicate that EGCG is effective in the prevention of UVA-induced ARPE19 cell damage.

### EGCG inhibits UVA-induced H_2_O_2_ production in ARPE19 cells

**Figure 4:**
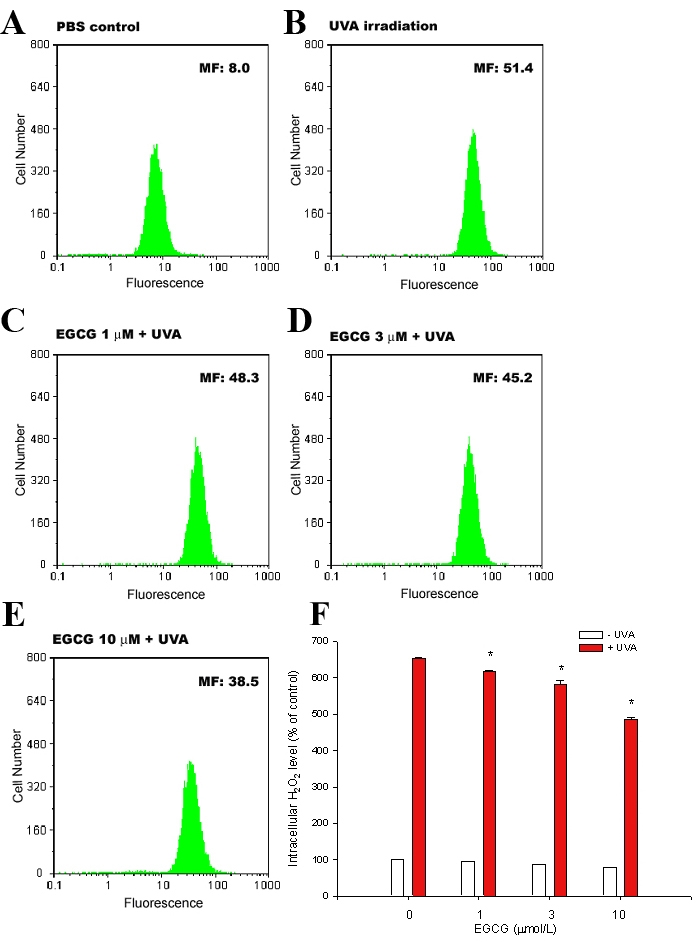
Effect of EGCG on intracellular H_2_O_2_ production of ARPE19 cells after UVA radiation exposure. ARPE19 cells pretreated with PBS (**A,B**) or EGCG for 2 h (**C-E**) were loaded with DHR 123. After being exposed to 20 J/cm^2^ UVA radiation (**B-E**), cells were collected and subjected to flow cytometric analysis. Results are presented as histograms of the number of cells versus fluorescence intensity. Each histogram represents three independent experiments. **F**: Quantitative analyses of intracellular H_2_O_2_ in ARPE19 cells (n=3). The asterisk indicates p<0.05 versus UVA-exposed cells without EGCG pretreatment.

Previous studies have shown that H_2_O_2_ is generated and is responsible for cell damage in cultured human skin cells during UVA irradiation [26,27]. We therefore tested if UVA radiation could increase intracellular H_2_O_2_ production and if EGCG would inhibit it in ARPE19 cells. The amount of intracellular H_2_O_2_ in ARPE19 cells was measured by using DHR 123, a dye that has been shown to react with H_2_O_2_ in the presence of peroxidase and is extensively used for the detection of intracellular H_2_O_2_ [27,28]. Flow cytometric analysis showed that mean fluorescence, i.e., intracellular H_2_O_2_ production, was increased about sixfold in UVA-exposed cells over unexposed control cells (Figure 4A,B). However, the increase of intracellular H_2_O_2_ was inhibited by the treatment of EGCG in a concentration-dependent manner (Figure 4C-E). In particular, treatment of cells with 3 μM and 10 μM of EGCG significantly inhibited intracellular H_2_O_2_ production (Figure 4F; p<0.05 compared with the UVA-irradiated culture without EGCG). The observation indicates that EGCG can prevent intracellular H_2_O_2_ production when ARPE19 cells are challenged with UVA irradiation.

### EGCG inhibits UVA-induced MAPK activation

**Figure 5:**
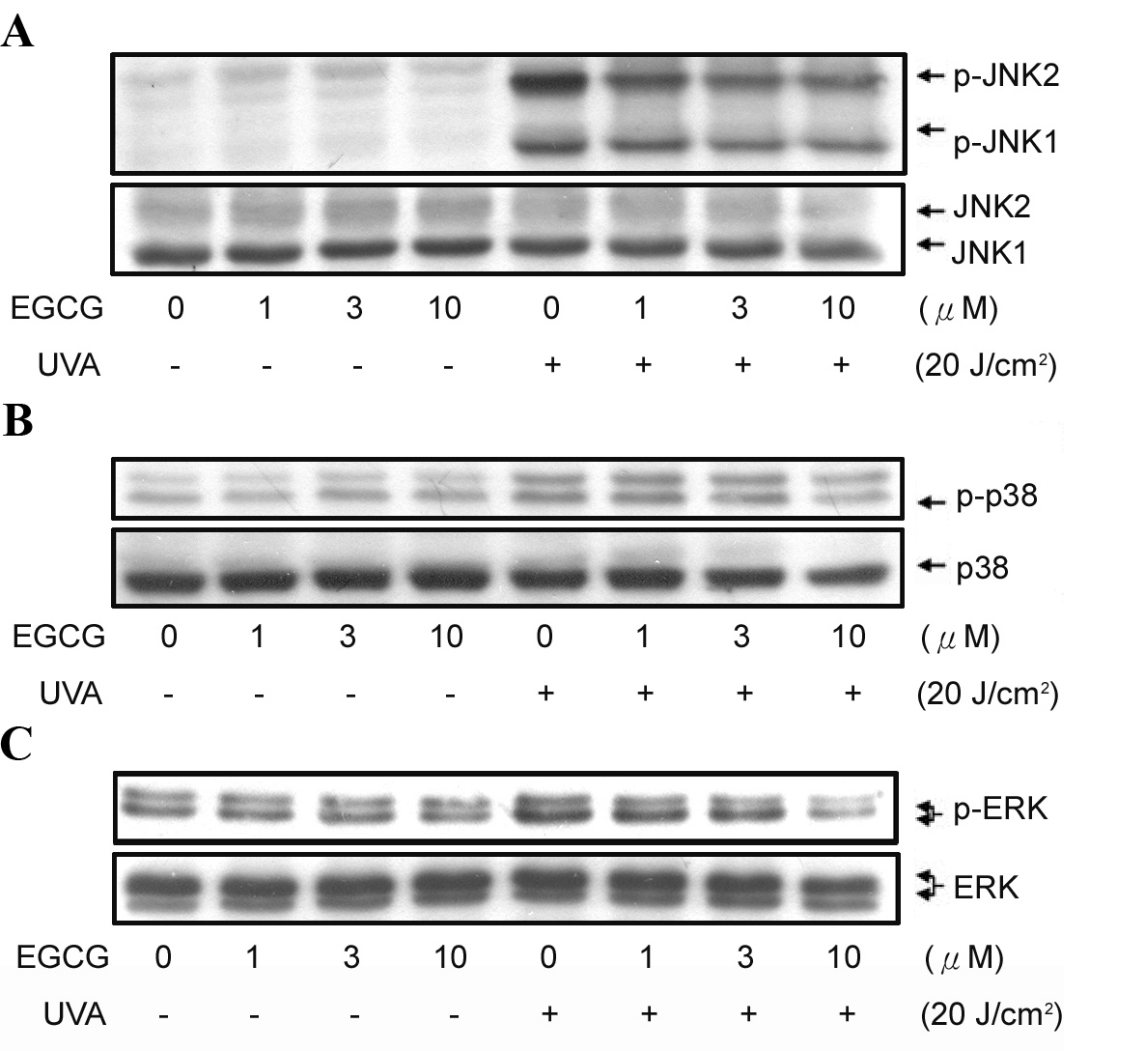
Effect of EGCG on UVA-induced JNK, ERK, and p38 phosphorylation. ARPE19 cells were preincubated with EGCG for 2 h then exposed to 20 J/cm^2^ UVA irradiation. After further incubation, cells were collected and lysates were analyzed by western blot analysis. Each result is representative of five to seven experiments.

UV irradiation has been shown to cause the activation of ERK, JNK, and p38 kinase [29], which may lead to RPE cell damage. In our experimental system, we also observed that ERK1/2, JNK, and p38 phosphorylation were increased in UVA-irradiated ARPE19 cells (Figure 5). However, ERK1/2 and JNK activations in ARPE19 cells were markedly reduced by the treatment of EGCG. UVA-induced p38 activation was only affected by EGCG at higher concentrations (Figure 5A-C, upper panels). Reprobing of the immunoblots with antibodies raised against total JNK, ERK1/2, and p38 demonstrated even loading of each sample (Figure 5A-C, lower panels). Our results demonstrate that EGCG affects MAPK activation and inhibits UVA-induced ARPE19 cell death.

### EGCG inhibits UVA-induced COX-2 activation

**Figure 6:**
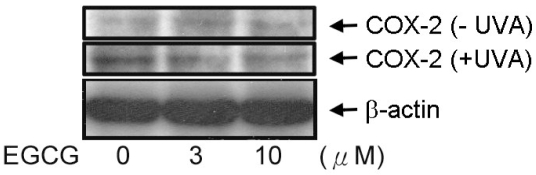
Effect of EGCG on UVA-induced COX-2 expression. ARPE19 cells were preincubated with EGCG for 2 h then exposed to 20 J/cm^2^ UVA irradiation. After further incubation, cells were collected, and lysates were analyzed by western blot analysis.

To find out what mechanism leads to cell death after UVA irradiation, we tested COX-2 expression in ARPE19 cells. Our observation was similar to those for other cell types [30,31] in that COX-2 expression, after exposure to UVA radiation, was increased in ARPE19 cells. The treatment of ARPE19 cells by EGCG reduced the expression of COX-2 (Figure 6, lower panel). The result suggests that inflammatory process may be correlated to UVA-induced ARPE19 cell death, and the reduction in COX-2 expression suggests that EGCG prevented the inflammatory process and death after UVA exposure.

## Discussion

Green tea contains many polyphenolic compounds that account for up to 15% of the dry weight of green tea leaves. Most of these polyphenols are flavanols, commonly known as catechins [32]. The EGCG and ECG forms account for about 36% and 24%, respectively, of all tea catechins [33]. Therefore, gallated catechins are major components of green tea catechins. At the molecular level, EGCG has been shown to have an additional phenolic hydroxyl group attached to its ring structure compared to ECG. The additional group is thought to have the most chemopreventive effect among all catechins in green tea [34,35]. Therefore, EGCG is well known as a scavenger of ROS in extracellular environments [36,37] and for its protective effect on many cell types against the damage of UV radiation [38]. However, whether EGCG possesses similar activity in retinal pigment epithelial cells against the damage of UVA radiation is not known. In the present work, we evaluated the ability of EGCG to protect against UVA-induced injury in RPE cells. We first demonstrated that EGCG could decrease UVA-induced cell death, H_2_O_2_ production, MAPK activation and expression of COX-2 in RPE cells. These effects will benefit the prevention of early AMD.

Regarding the possible mechanisms by which EGCG inhibits UVA-induced RPE cell death, our results showed that it may act through its antioxidative and antiinflammatory effects (Figure 4, Figure 5, and Figure 6). The biologic effects of UVA are primarily mediated by the generation of ROS, including singlet oxygen, superoxide anion, hydrogen peroxide, and hydroxyl radical. These active oxygen species mediate many damaging effects. For example, hydrogen peroxide could increase the apoptosis of RPE cells. In our study, we found that UVA-induced H_2_O_2_ production was decreased by pretreatment with EGCG. This inhibition, in turn, contributes to the protective effect of EGCG onUVA-induced RPE cell death. However, we also found that UVA-induced H_2_O_2_ production cannot be completely inhibited by EGCG treatment. This may be the reason EGCG could not completely reverse UVA-induced cell death (Figure 3 and Figure 4).

UV irradiation has been reported to upregulate expression of transcription factors [39,40], which is mediated by the sequential activation of cytoplasmic protein kinases. To date, three structurally related but biochemically and functionally distinct MAPK signal pathways have been identified, including ERK1/2, JNK, and p38 [41]. Activation of MAPK is essential for ROS (e.g., hydrogen peroxide) -induced apoptosis in RPE cells [42]. In our study, we found that EGCG inhibited UVA-induced ERK1/2 and JNK activation at lower concentrations (1–3 μM) than p38 activation (10 μM; Figure 5). It is generally accepted that ERK activation is essential for cell survival, whereas activation of JNK and p38 is thought to play an important role in cell death signaling. Thus, we suggest that inhibition of JNK and p38 would contribute to the protective effect of EGCG against UVA-induced cell death.

The major role of COX-2 in angiogenesis is thought to be induction of the synthesis of prostagladins, which may stimulate the expression of proangiogenesis factors [34,35]. Additionally, COX-2 may directly affect angiogenesis by modulating the expression of vascular endothelial growth factor ligand and receptor [36]. Selective COX-2 inhibitors attenuated choroidal neovascularization formation in an animal model of angiogenesis [37-39], providing evidence that COX-2 is implicated in CNV. We observed an increase in the expression of COX-2 in UVA-irradiated RPE cells. Moreover, we found that EGCG could decrease COX-2 expression. This suggests that EGCG may possess bioactivities that prevent the formation of choroidal neovascularization. In addition, MAPK activation is correlated with COX-2 expression in many cell types as reported in previous studies [43,44]. Therefore we also suggest that the inhibition of MAPK phosphorylation may partially contribute to the inhibition of COX-2 expression and to the protective effect of EGCG.

In the previous report [45], the doses used were approximately equivalent to the consumption of 2–53 cups of green tea (185 mg of EGCG per cup) by a 70 kg person. In animal study (male CF-1 mice), no acute toxicity was observed after single-dose administration of up to 2000 mg/kg intragastric infusion (i.g). A linear increase in the plasma concentration of EGCG was observed 50 and 180 min after administration of 50– 2000 mg/kg i.g. EGCG to male CF-1 mice. The concentration of total and unconjugated EGCG in the plasma ranged from 0.03 to 4.17 µg/ml and 0.01 to 0.37 µg/ml, respectively [45]. In a study by Henning et al. [46], the maximum plasma total flavanol (EGC+EC+EGCG+ECG) concentration after Poly E intake was 3.2 μM. In the present study, we examined the protective effects of EGCG at 1–10 μM. Therefore we predict the concentration of 1–10 μM could reach the ocular organ after the consumption of green tea extract or purified EGCG. Moreover, the concentrations we used can possibly be achieved if their bioavailabilities and several concentrations can be improved. Therefore, we suggest that EGCG could supply protective effects in eyes after intake of EGCG or green tea.

In summary, our study provides the first evidence that EGCG is a protective agent for RPE cells after exposure to UVA radiation. Our results demonstrate that EGCG inhibits UVA-induced H_2_O_2_ production, MAPK activation, and expression of COX-2. Moreover, EGCG increases ARPE19 cell survival after exposure to UVA radiation. This suggests that EGCG is effective in preventing ARPE19 cells from UVA-induced damage and may be suitable to be further developed as a chemoprotective agent for the primary prevention of early AMD, especially for patients after cataract surgery. In addition, our results imply that green tea catechins can have a positive effect and can play an important role in the prevention of oxidation or UVA-induced ocular disorders.
